# Construction and characterization of nano-oval BaTi_0.7_Fe_0.3_O_3_@NiFe_2_O_4_ nanocomposites as an effective platform for the determination of H_2_O_2_

**DOI:** 10.1038/s41598-023-36076-6

**Published:** 2023-06-03

**Authors:** Ali B. Abou Hammad, Hend S. Magar, A. M. Mansour, Rabeay Y. A. Hassan, Amany M. El Nahrawy

**Affiliations:** 1 Solid State Physics Department, Physics Research Division, National Research Centre, 33 El Bohouth St., Dokki, Giza, 12622 Egypt; 2 Applied Organic Chemistry Department, National Research Centre (NRC), 33 El-Bohouth St., Dokki, Cairo, 12622 Egypt; 3 Biosensors Research Laboratory, Zewail City of Science and Technology, 6Th October City, Giza, 12578 Egypt

**Keywords:** Energy science and technology, Materials science, Nanoscience and technology, Physics

## Abstract

Talented di-phase ferrite/ferroelectric BaTi_0_._7_Fe_0_._3_O_3_@NiFe_2_O_4_ (BFT@NFO) in oval nano-morphology was chemically synthesized using controlled sol–gel processes and calcined at 600 °C. The effects of shielding using NiFe_2_O_4_ (NFO) nanoparticles on the microstructure, phase transition, thermal, and relative permittivity of BaTi_0_._7_Fe_0_._3_O_3_ (BTF) nano-perovskite were systematically explored. X-ray diffraction patterns and Full-Prof software exhibited the forming of the BaTi_2_Fe_4_O_11_ hexagonal phase. TEM and SEM images demonstrated that the coating of BaTi0._7_Fe_0_._3_O_3_ has been successfully controlled with exquisite nano-oval NiFe_2_O_4_ shapes. The NFO shielding can significantly promote the thermal stability and the relative permittivity of BFT@NFO pero-magnetic nanocomposites and lowers the Curie temperature. Thermogravimetric and optical analysis were used to test the thermal stability and estimate the effective optical parameters. Magnetic studies showed a decrease in saturation magnetization of NiFe_2_O_4_ NPs compared to their bulk system, which is attributed to surface spin disorder. Herein, characterization and the sensitive electrochemical sensor were constructed for the evaluation of peroxide oxidation detection using the chemically adjusted nano-ovals barium titanate-iron@nickel ferrite nanocomposites. Finally, The BFT@NFO exhibited excellent electrochemical properties which can be ascribed to this compound possessing two electrochemical active components and/or the nano-ovals structure of the particles which can further improve the electrochemistry through the possible oxidation states and the synergistic effect. The result advocates that when the BTF is shielded with NFO nanoparticles the thermal, dielectric, and electrochemical properties of nano-oval BaTi_0.7_Fe_0.3_O_3_@NiFe_2_O_4_ nanocomposites can be synchronously developed. Thus, the production of ultrasensitive electrochemical nano-systems for the determination of hydrogen peroxide is of extensive significance.

## Introduction

In recent years, perovskite materials have been well studied. The most prominent ABO_3_ ferroelectric materials have attracted considerable attention as catalysts due to their geometric and electronic stability, higher dissolution resistance in aqueous and non-aqueous solutions, and cost-efficiency. There is rare existence of crystalline multiferroic compounds in which ferromagnetism and ferroelectricity coexist at room temperature^1^. Due to their potential application in the developing field of information storage, spintronics, and multiple-state memory storage devices, such compounds are currently under intensive study^2,3^. Enormous attempts were made to improve the room temperature ferromagnetism and ferroelectricity in perovskite ceramics. Various approaches are underway to explore the possibility of synthesizing materials with superior multiferroic efficiency. Another possible method of magnetic doping is of TM (transition metal) ions (Fe^3+^, Co^2+^, Ni^2+^, Mn^2+^, etc.) through ferroelectric materials^4,5^.

A major class of ferroelectric material that shows a piezoelectric effect and a high dielectric permittivity for technological applications, such as transducers, surface acoustic waves (SAW), and ferroelectric random-access memories (FeRAM) devices^6^. In the composition of perovskite ABO_3_, both cations (A and B), whereas the A atoms are greater than the B atoms. The ionic radii of Fe^3+^ (0.645 Å) are larger than Ti^4+^ (0.605 Å), whereas Ba^2+^ has larger ionic radii (1.35 Å). BTO has a general ABO_3_ configuration in which Ba^2+^ and O^2−^ ions create a close-packed cubic lattice with Ti^4+^ ions occupying the oxygen (O^2−^) generated octahedral holes. The BaTiO_3_ structure has a three-dimensional array of TiO_6_ corners sharing octahedral with Ba^2+^ ions in the 12-fold cavities between the polyhedra. The arrangement of the atoms is known as the close-packed array of the A^2+^ and O^2−^ ions together^7^. In addition to its structure and size, the properties of BTO depend on its chemical composition. Doping with equivalent elements, perovskite material shows interesting electrical characteristics for the BaTiO_3_ based on the chemical states of the components and the surface chemistry of the specimens. However, the major phenomenon of these nanomaterials is the partial substitution of the cations at the A and B sites of ABO_3_ as well as their ability to preserve the stability in the crystal structure of uncommon mixed oxidation states^8,9^. An interesting aspect of Ba, Ti, Fe, and O ions in bulk materials has been discussed concerning both electronic deficiency and surface toxicity. Several recent studies have been addressed based on photoelectron spectroscopy for the chemical analysis (ESCA) of calcined BaTiO_3_ nanomaterial doped with Fe^3+^ ions^9–12^. ESCA or XPS is a surface morphological technique that can be used to obtain information on the chemical state or valence state and the electronic information at the core level for the constituent element^9–12^. Pure BaTiO_3_ has lower ionic conductivity and acts as an insulator at room temperature. The defect model indicates that the substitution of the Ti-site by the acceptor impurities is the reason for the formation of charge carrier and oxygen vacancies that increase both the ionic and electronic conductivity. The dominant charge carrier depends on the synthesis conditions and the amount of unintentional impurities of the acceptor and the donor. In BaTiO_3_ ceramics, Fe^3+^ ions usually replace Ti^4+^, where Fe^3+^ ions are considered to have a 3 + valence. Therefore oxygen vacancy should be created to preserve the charge neutrality of the perovskite structure (BaTi_1-x_Fe_x_O_3-δ_, δ-oxygen vacancy). Abdel Aal et al. 2014 successfully prepared BaTi_1-x_Fe_x_O_3_ by sol–gel, where the trivalent ions (Fe^3+^) replace the tetravalent ions Ti^4+^ and create oxygen vacancy to maintain the charge neutrality of the compound^13^.

Understanding the impact of the substitution of TM on structural, physical, and microstructural properties will be a significant step in the explanation of these properties^14–16^. Nickel ferrite, as an inverse spinel-type with molecular form (NiFe_2_O_4_), offers ferrimagnetism behavior that originates from trivalent (Fe^3+^) and the divalent (Ni^2+^) cations distribution in B and A locations. In the NiFe_2_O_4_ structure, (Ni^2+^) ions are in B-sites, and trivalent (Fe^3+^) ions are equally divided between both (A and B) sites^17^. NiFe_2_O_4_ nanoparticles are used in various applications because of their extraordinary properties including lower toxicity, small magnetic anisotropy, excellent electrochemical activity, elevated electrical conductivity, and lower Curie temperature. Usually, their properties are constructed on synthesis manner, size scale, and the grain shape of the NiFe_2_O_4_ material^18–20^.

Abou Hammad et al. 2020 have prepared Perovskite/spinel nanocomposites by advanced sol–gel, where they prepared the spinel ferrite CoFe_2_O_4_ powders then they coated it by ZnTiO_3_ through dispersing the CoFe_2_O_4_ nanopowders in ZnTiO_3_ solutions and drying the solution with slow rate to enable building up the shell layer (ZnTiO_3_)^21^.

On the other hand, the development of reliable and sensitive sensors for hydrogen peroxide detection by an easy method using a low-cost way is important. Many analytical techniques have been carried out for H_2_O_2_ detection, such as chemiluminescence^22^, fluorescence^23^, spectrophotometry, chromatography, and electrochemistry^24–27^. Electrochemical detection is distinctive due to simplicity, selectivity, sensitivity, and low cost^28–34^. The electrochemical biosensor is an ideal detection tool for the fast, sensitive, and accurate detection of drugs^35^, biological samples, and the diagnosis of diseases^36^. All of these interesting properties of the electrochemical sensors depend on the type of electrode materials. Hence, it is necessary to provide new nanomaterials that have electro-catalytic properties to evaluate an effective electrochemical sensor platform(s). When controlled the energy band of BTO-nanostructures, the optoelectronic and catalytic activity will be developed to meet the advanced applications.

Therefore, in this work, the piezoelectric BTO shielded with ferrites will acquire a partial semiconducting property hybridized with efficient electric, catalysts, and electrochemical performance can lead to significantly enhanced catalysis operation according to the piezo-nanomagnetic catalysis effect.

Hence, the study of spinel ferrites as a shield layer for the perovskite is considered of scientific and industrial enormous importance because of their new properties, significant magnetic and electrical properties, excellent chemical stability, and wider applications. The present study investigates the impact of doping of NFO NPs with various concentrations on BTF nanoperovskite. Also, the ability of nano-oval BaTi_0.7_Fe_0.3_O_3_@NiFe_2_O_4_ nanocomposites as an effective platform for the determination of H_2_O_2_ is investigated. The structural, thermal, optical, dielectric, and electrochemical properties of the nanomaterials have been studied. Due to the high electrocatalytic activity properties of doping of NiFe_2_O_4_ ions with various concentrations on the BTF materials, there are modifications on the screen-printed electrodes surface, and the direct electrochemical oxidation of peroxide was studied using cyclic voltammetry and chronoamperometric techniques. The prepared nanostructures showed high sensitivity toward peroxide detection, which can be effective in the enzymatic biosensor fields.

## Materials and methods

### Materials

For the synthesis of Ba Fe_0.3_Ti_0.7_O_3_and NiFe_2_O_4_phases, high grade titanium (IV) isopropoxide Ti[OCH(CH_3_)_2_, barium acetate (Ba(CH_3_COO)_2_,99.9%), iron nitrate Fe(NO_3_)_3_. 9H_2_O (99.9%),Ni(NO_3_)_2_.6H_2_O (98%), acetyl acetone (AcAc, C_5_H_8_O_2_, and nitric acid(HNO_3_) were used as a raw materials. Potassium ferrocyanide, potassium ferricyanide, potassium chloride, potassium dihydrogen phosphate, potassium monohydrogen phosphate, hydrochloric acid (HCl), sodium hydroxide (NaOH), and hydrogen peroxide were purchased from Sigma-Aldrich.

### Synthesis of multiferroic BaTi_0.7_Fe_0.3_O_3_ shielded with NiFe_2_O_4_ nanocomposites

First, in the NiFe_2_O_4_ coating layer, the stoichiometric amounts of Fe(NO_3_)_3_·9H_2_O/H_2_O and Ni(NO_3_)_2_·6H_2_O/H_2_O are mixed in (1:2) ratio with a dissolved citrate acid (CA) under stirring. After hard stirring for 1 h, the NFO sols aged at room temperature (RT) for 5 h before coating BTF nanocrystallites.

Iron barium titanate **(**BTF) shielded with nickel ferrite (NiFe_2_O_4_, NF) was synthesized by the adapted sol–gel process. The sol–gel process allows the formation of multiferroic magnetoelectric and the controlled coexisting of magnetic and electric phases in one structure.

**Figure 1:**
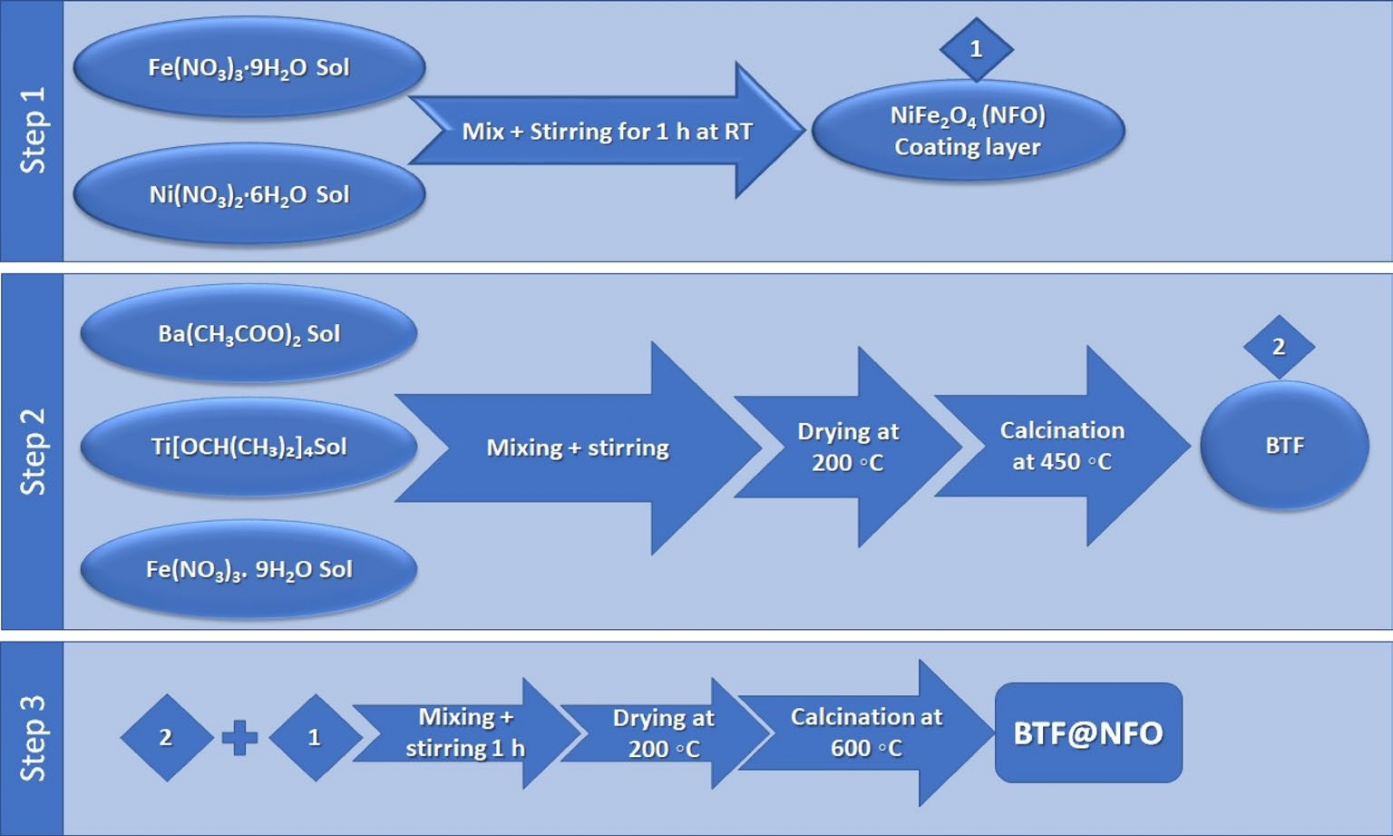
Synthesis steps of multiferroic BaTi0.7Fe0.3O3 shielded with NiFe_2_O_4_ nanocomposites.

Barium titanate iron nanocrystallites were prepared by sol–gel process using the evaluated amounts of barium acetate, titanium (IV) isopropoxide, and iron nitrate as a source of Ba, Ti, and Fe, respectively. Acetic acid (HAc) and acetylacetone (AcAc, C_5_H_8_O_2_) were used as suitable solvents. Titanium (IV) isopropoxide was dissolved in acetylacetone, while both barium acetate and iron nitrate were dissolved in acetic acid/water. The solutions were mixed, stirred for 1.30 min, and dried at 200 °C. Further, thermal treatment was done at 450 °C for 2 h, after which barium titanate iron powder was shaped (Fig. 1).

For BTF/(x) NF (x = 0, 1, 3, and 5), the resulting BTF nanopowder, after calcination at 450 °C, was added to the solution of NiFe_2_O_4_ according to the desired weight percent. The obtained solution was stirred for 1 h at 80 °C. The solution dried at 200 °C and turned into a viscous brawn gel, and started yielding xerogel form after evaporating the aqueous media. Finally, the obtained xerogels were calcinated at 600 °C. The constructed nanocomposite samples were labeled with symbols 0B, 1B, 2B, and 3B.

### Material characterizations

The prepared perovskite-NiFe_2_O_4_ nanocomposites were characterized using the powder X-ray Diffraction (XRD-XPERT) through (Cu Kα) radiation. The profiles of XRD for the nano-oval BaTi_0.7_Fe_0.3_O_3_@NiFe_2_O_4_ nanocomposites were then refined using fixing the instrumental and shape parameters by Full-prof software (the Pseudo-Voigt fitting-model) and geometrical assembly was drawn by the VESTA: win64 software^37,38^. Transmission electron microscopy (High-resolution-TEM, (FEI; Tecnai- T20)) was used.

Thermogravimetric analysis measurements (TGA) in flowing imitation air (flow rate 20 ml/min, heating rate 50 K/min) of the pre-dried 200 °C solutions was achieved using Netzsch (STA 449 system), with a heating rate of (10 °C/min).

The optical bandgap of the nano-oval composites was evaluated using UV–Vis-diffuse reflectance spectroscopy (JASCO: V550) and also the optical bandgap can be evaluated through Tauc Plot manner.

Dielectric capacities were achieved using (LCR METER-IM3536) by a frequency range of 4 Hz–8 MHz. The samples were pressed in a distinct die (diameter = 10 mm) to procedure tablets with a thickness of ~ 1.2 mm.

### Electrochemical analyses

**Figure 2:**
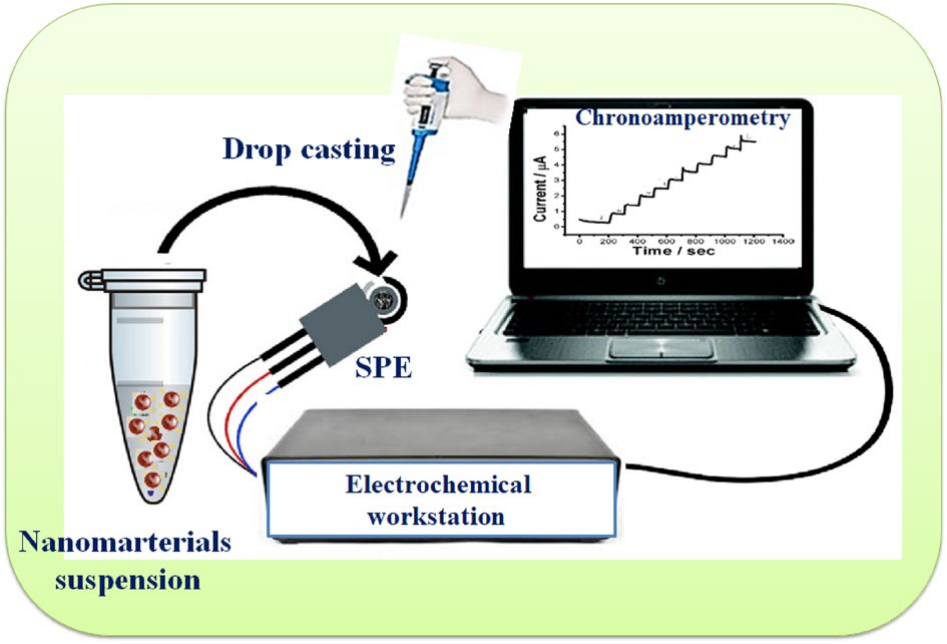
Steps of SPEs modification with 0B, 1B, 2B and 3B nanostructures before testing their electrochemical performance.

Screen printed electrodes (SPEs) were used as the sensing platform for testing the electrochemical properties of the newly synthesized nanocomposites. Electrochemical techniques (cyclic voltammetry (CV), and electrochemical impedance spectroscopy (EIS)) were conducted using CHI-potentiostat. For electrode surface modification with the nanomaterials, 5.0 mg of the synthetic nanocomposite was added to 1.0 ml double distilled water and then ultra-sonicated for up to 30 min to produce a homogenous suspension. Then 30 μl of the suspended solution was dropped on the SPE surface and left to dry. For SPE characterization, CV and EIS measurements were carried out in a solution containing 5 mM of the ferricyanide [Fe (CN)_6_]^3‑/4‑^ and 0.1 M KCl. For H_2_O_2_ detection chronoamperometric measurements were carried out in a PBS buffer. The following figure (Fig. 2) shows the simple modification method of SPEs with the 0B, 1B, 2B, and 3B nanocomposites for testing their electrochemical performance^34^.

## Results and discussion

### phase identification (XRD)

**Figure 3:**
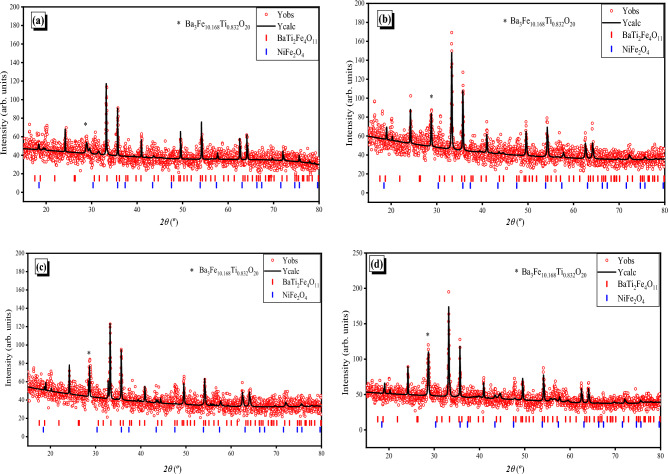
XRD patterns of (**a**) BaTi_0.7_Fe_0.3_O_3_ shielded by NiFe_2_O_4_ NPs with concentrations (**b**) 1, (**c**) 3, and (**d**) 5 mol., calcined at 600 °C.

The formation of nano-oval BaTi_0.7_Fe_0.3_O_3_ shielded (1, 3, 5 mol.%) NiFe_2_O_4_ nanocomposites are confirmed by the XRD profile/ FullProf as revealed in Fig. 3a–d. Obviously, the growth of hexagonal BaTi_2_Fe_4_O_11_ and cubic NiFe_2_O_4_ phases indicated that the lower calcination core BTF nanoparticles had been reacted with the shield NiFe_2_O_4_ for new arrangement within the BaTi_0.7_Fe_0.3_O_3_@ NiFe_2_O_4_ complex. Figure 3a, d show a presence of a secondary phase ( monoclinic phase of Ba_3_Fe_10.168_Ti_0.832_O_20_). The monoclinic phase can disappear with increasing the calcination temperature higher than 800 °C. The calcination of nao-oval composites at 600 °C exhibited a well crystalline degree, with sharp and high intensity peaks. Additionally, the average crystallite size (ca. 21–23 nm) of the BaTi_0.7_Fe_0.3_O_3_ shielded NiFe_2_O_4_ nanocomposites, which was calculated from the Scherrer formula^20,21,42^. It is declared that the BTF is well shielded with the NiFe_2_O_4_ and has adequate stabilization.

The Rietveld analysis and the lattice parameters of the phases were given in Table 1. The XRD results indicated the good shielding of BTF nanoparticles in NFO NPs matrix, produced from the modification of BTF nanoparticles using NFO and calcination at 600 °C.

**Table 1:** the lattice parameters for the formed phases.

Sample	BaTi_2_Fe_4_O_11_ (hexagonal)					NiFe_2_O_4_ (cubic)			
	a = b	c	Vol	α = β	γ	a = b = c	Vol	α, β, γ	*ρ*_*x*_
0B	5.8432	13.604	402.253	90	120	8.3370	579.468	90	5.373
1B	5.8474	13.602	402.77	90	120	8.3408	580.261	90	5.366
2B	5.8462	13.612	402.938	90	120	8.3382	579.676	90	5.371
3B	5.8472	13.609	402.951	90	120	8.3406	580.219	90	5.366

The X-ray density can be obtained from applying the following equation.^43–45^:

$${\varvec{\rho}}_{{\varvec{x}}} = \frac{{8{\varvec{M}}}}{{{\varvec{N}}_{{\varvec{A}}} {\varvec{V}}}}$$

M is the molecular mass, V is the volume, and NA is the Avogadro number.

### Morphological analysis using TEM

**Figure 4:**
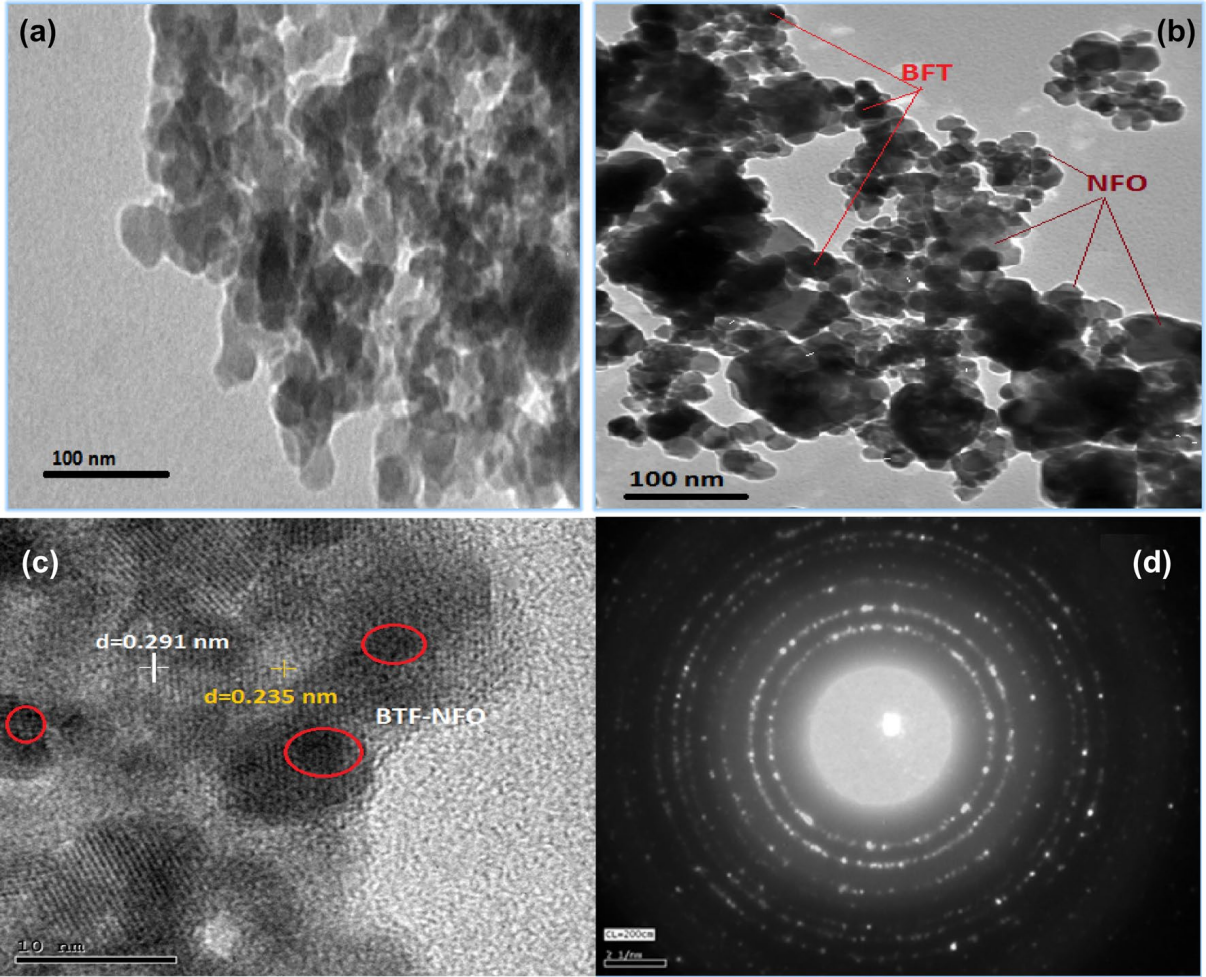
TEM images of nano-oval BaTi_0.7_Fe_0.3_O_3_ and shielded with 5 NiFe_2_O_4_ nanocomposites, calcined at 600 °C.

The particle nature of nano-oval BaTi_0.7_Fe_0.3_O_3_ shielded with NiFe_2_O_4_ nanocomposites was investigated using transmission electron microscopy (TEM). Figures 4 showed the TEM images of the nano-oval BaTi_0.7_Fe_0.3_O_3_ shielded with 5 NiFe_2_O_4_ nanocomposites. The results confirmed that the samples have nano-spherical and nano-ovals morphology (with the average size of 12 − 25 nm in diameter).The microstructures of the shielded ovals nanocomposite indicated that the BTFO phase is obviously described by regular grains, which are homogeneously distributed and connected with the NFO matrix. The samples showed a significant clustering to affect the XRD intensity. The occurrence of certain accumulated dark spots in the TEM images is due to the shielding of BTF with NFO and the interaction among magnetic NFO nanoparticles with the higher surface energies of the BTF. The crystal lattice displayed no clearly lattice deficiencies with clear boundaries. The internal distance between the two neighboring lattice planes is evaluated to be 0.292 nm associated with the main XRD peak of BTF and 0.235 nm associated with the main XRD peak of BTF@NFO, which confirmed the successful shielding with the NFO nanoparticles. The TEM results indicated that the NiFe_2_O_4_ is highly shielded and well adhesion with the BTF nanoparticles. Moreover, the TEM data is supporting the XRD results. TEM images are evidence that the magnetic particle aggregation lies in the nanometric region.

### Thermal study

The thermogravimetric test TGA is a technique that records the weight variations when a solid is warmed up at a uniform speed to evaluate its thermostability and volatile ingredient fraction. Since the object is processed under different conditions, the TGA technique falls under the category of thermal analysis^40^. The approach allows simultaneous measurement of the temperature, time, and mass of a sample in a managed flexible setting. These assessments are based on the fluctuation in weight of the material as a response to warming a condition^46,47^. Therefore, a certain weight amount will be destroyed and evaluated as volatiles or decomposed, and this loss is detected with precision balance. The material is loaded in a cylinder pan built specifically for this testing. Temperature variations are determined by a tailored temperature program, which may include isothermal and ramp stages with varying warming levels^39^. The temperature of the cuvette is monitored using thermocouples in connection with it. For weight monitoring, the cuvette is normally put on a test stand which is attached to a mass-sensitive instrument. The sample holding cuvettes of a TGA device can be of various forms and compositions. They must be able to properly keep the sample, inert to it, and the heating conditions. Alumina, platinum, and aluminum are the most common materials used to make testing cuvettes^40^. An exhaust gas flowing into the oven creates an atmosphere that can be neutral, such as nitrogen or argon, oxidative, such as air or oxygen, or reductive, such as a mixture of hydrogen and nitrogen. The amount of moisture inside the oven could range from dry to saturated^40^.

In this work, thermogravimetric analysis was carried out by TA instruments Lab Q5 in a nitrogen gas environment at temperatures ranging from 25 to 900 °C at a 10 °C/min heating rate. The TA Instruments Q50 TGA monitors the change in sample weight as a function of temperature. It employs delicate microbalance as well as precise heating management. The specimen mass limit is 1 g with an accuracy of 0.1 g.

**Figure 5:**
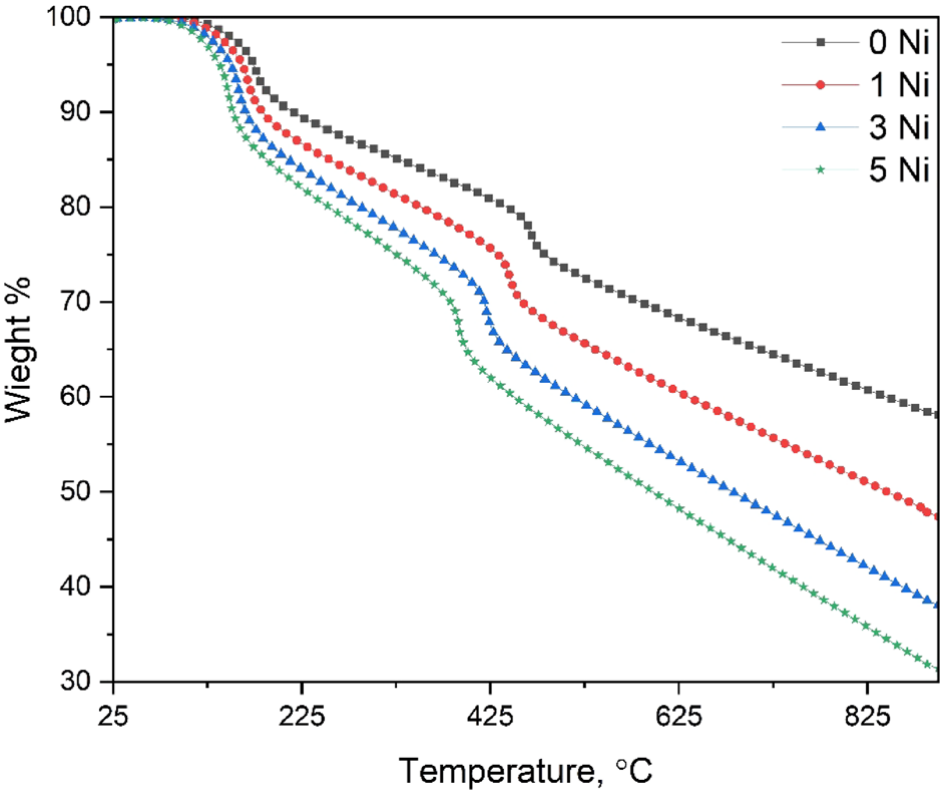
TGA curves of the prepared nanocomposites in the range of ambient to 900° C in an N_2_ atmosphere with a heating rate of 10 °C/min.

The TGA curves of nanocomposites in the N_2_ climate with a heating rate of 10 °C/min are shown in Fig. 5. The behavior shows two main decomposition reactions; the first one is mostly due to the dehydration and evaporation of organic components^44,45^between (125–205 °C), (117–189 °C), (110–177 °C) and (97–167 °C) for the samples, respectively. The mass loss of 0, 0.1, 0.3, and 0.5 NiFe_2_O_4_ is approximately 9, 11, 13, and 14% of the initial weight, respectively. Because the range of events that occur in the first reaction decreases, the NiFe_2_O_4_ nanoparticle shells are not favorable to the thermal stability of the nanocomposites^45,48^.

The second stage situated in the range of (457–481 °C), (434–460 °C), (412–434 °C), and (282–401 °C) for, 0.1, 0.3, and 0.5 NiFe_2_O_4_, respectively. This stage reflects the decomposition of the nanomaterial^43,48^. The samples (0.1, 0.3, and 0.5 NiFe_2_O_4_) loss was about 25, 30, 34, and 36% of their initial weight, respectively. The addition of NiFe_2_O_4_ nanoparticle shells led to a decrease in the range of occurrence of the process.

### Diffuse reflectance

Diffused reflectance is the phenomenon resulting from reflection, refraction, diffraction, and absorption orientated in all directions. The discrete light absorption as well as the size-dependent are developed due to the confinement of quantum size^19^. In the case of nanocrystalline semiconductors, linear (one exciton per particle) and nonlinear (multiple excitons per particle) properties arise as a result of the transition from an electron and an electron hole to discrete (quantized) electronic shells. Optical absorption of the nanosized oxides is influenced by nonstoichiometric defects which are size-dependent. In nanostructured oxides, the point defects concern the presence of dopant and/or the vacancies of cation or oxygen. In proportion to the number of defects, gap states are introduced by vacancy defects.

**Figure 6:**
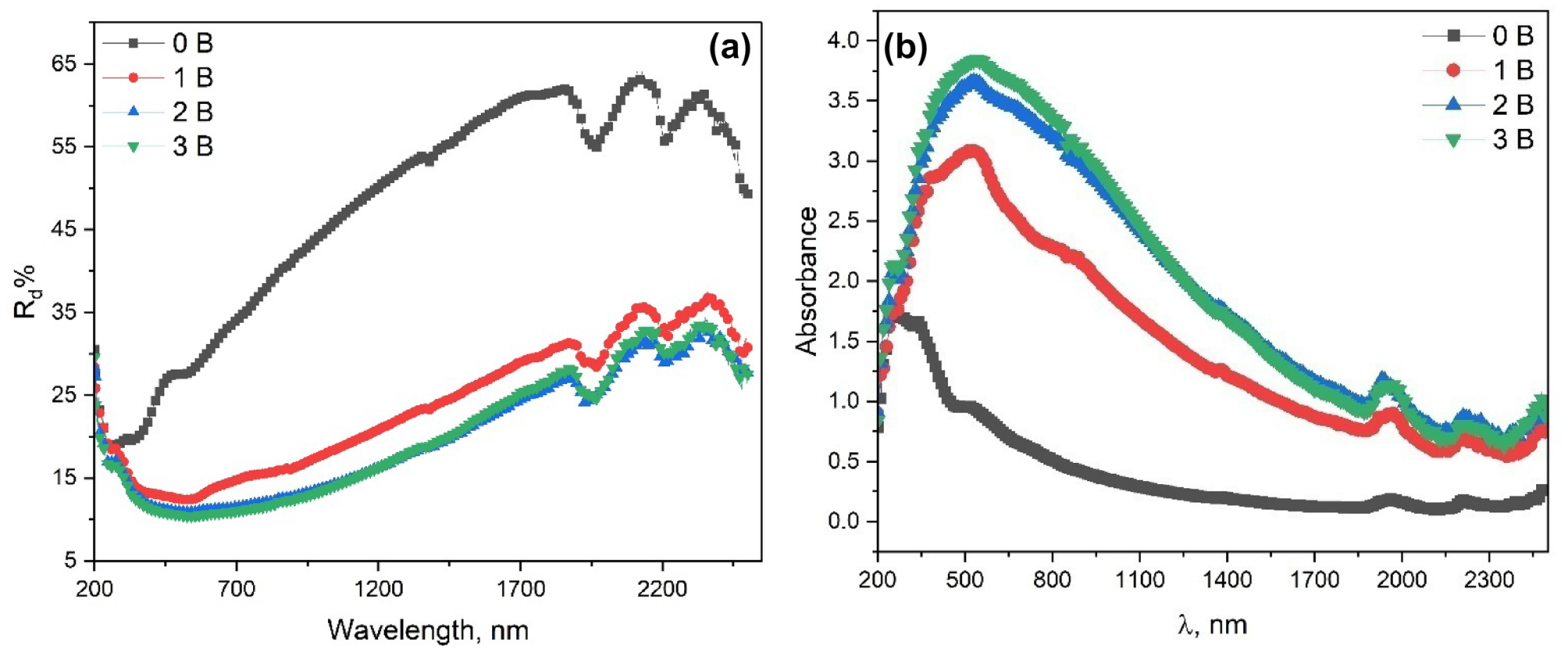
(**a**) Diffuse reflectance and (**b**) absorptance spectra of BaTi_0.7_Fe_0.3_O_3_/(0, 0.1, 0.3, 0.5) NiFe2O4 nanocomposites in the wavelength range of 200 to 2500 nm.

Diffuse reflectance was used to study the optical properties of the prepared BaTi_0.7_Fe_0.3_O_3_/(0, 1, 3, 5) NiFe_2_O_4_ (BTFO/NFO ) nanocomposites in the wavelength range of 200 to 2500 nm, as shown in Fig. 6. The diffuse reflectance of all samples increases with wavelength increase until about 1870 nm, where the interference takes place.

Furthermore, in Fig**.** 6a, the increase in NiFe_2_O_4_ leads to a decrease in diffuse reflectance values. Previously^49^, it was observed that the absorption spectrum of the BTO exhibits a sharp absorption edge above 3 eV and peaks at 3.51 eV roughly taken as the value of the band gap, that is, 3.51 eV. This edge corresponds to the band-to-band absorption scenario in the 'extrinsic band insulator of the tetragonal BaTiO_3−δ_^49^. In that case, the oxygen deficiency content δ, which is quite small, can be qualitatively reflected from a long tail below the rising edge in the range of photon energies between 2 and 3 eV^49^. In the current work, the absorption spectrum of the BTFO becomes broadening (Fig. 6b), which spans a wide range of photon energies from the ultraviolet region to the near-infrared one. This spectrum can be spectroscopically de-convoluted into two Lorentzian components as shown in Fig. 6b, whose peaks are at about 340 nm and 520 nm, respectively. The first component may be attributed to a combination of multiple absorption processes to donor levels in the forbidden band^49^.

However, the second component is due to band-to-band absorption for hexagonal BTFO, i.e., Fe-doped hexagonal BTO, with a band gap Eg 2.7 eV smaller than that of the undoped tetragonal BTO (Eg ≈ 3.51 eV)^49^. In addition, the red shift observed for UV–VIS absorption spectra is a direct consequence of the change of the band structure from the tetragonal BTO crystal to the hexagonal BTFO one, which is caused by substituting the Fe ion for Ti^4+^ to create Fe ions with an oxidation state of about 3.92 and positively charged oxygen vacancies as the electron trapping centers.

The method of Kubelka and Munk (K–M) was employed to determine the optical band gap (Eg) of the BTF/NiFO materials, which is based on the conversion of the diffused reflectance measurements. The Kubelka–Munk equation is given below for a specific wavelength:

1 $$F\left( {R_{d} } \right) = \left( \frac{k}{s} \right)\left( {1 - R_{d} } \right)^{2} /2R_{d}$$

where F(R_d_) is the K–M function or the absolute reflectance of the material. In reflectance measurements, barium sulphate (BaSO_4_) is used as a standard sample. ‘k’ is the coefficient of molar absorption and ‘s’ is the scattering coefficient. The relation between the optical band gap and the absorption coefficient of semiconductor oxide materials was determined by Wood and Tauc in the case of a parabolic band structure. According to them, the following equation is based on the optical band gap in absorption and photon energy^50^:

2 $$F\left( {R_{d} } \right)h\nu = C\left( {h\nu - E_{g} } \right)^{n}$$

where ‘α’ is the absorption coefficient of the samples and ‘hυ’ is the photon energy. C is the constant factor, E_g_ is the optical band gap, and ‘n’ is a constant correlated with various types of electronic transition (n = 1/2 for a direct allowed transition, n = 2 for an indirect allowed, n = 3/2 for a direct forbidden, and n = 3 for an indirect forbidden transition) as previously reported in the literature^19,51^.

**Figure 7:**
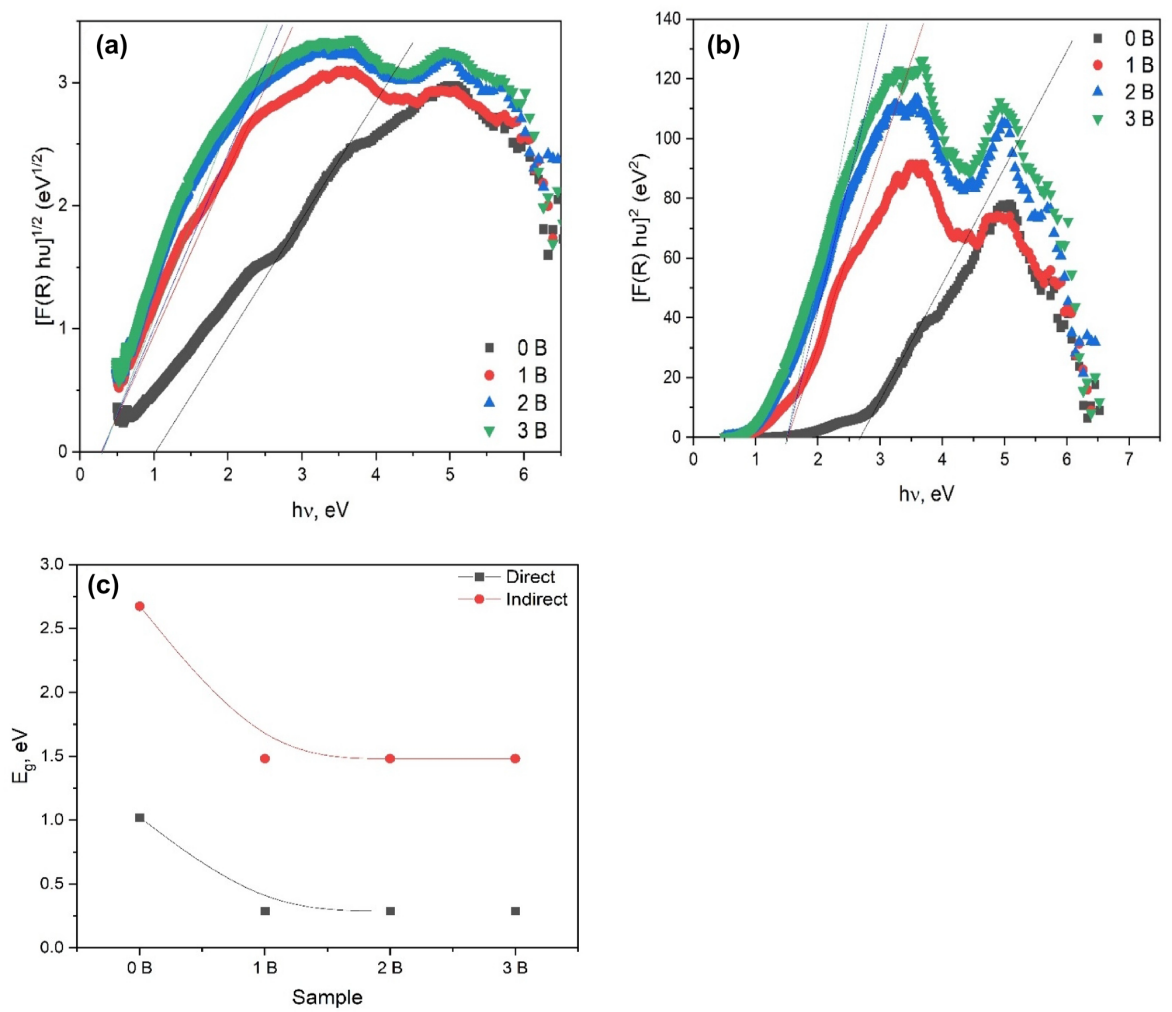
Plot of [F(R) hυ]^n^ and hυ for (**a**) n = 2 and (**b**) n = 1/2, and (**c**) the behavior of energy band gap with the Ni content in both direct and indirect cases.

The BTFO/NiFO nanocomposites show an optical absorption spectrum caused by the direct and indirect electronic transition with a higher probability of the direct one. In the direct mechanism, electrons in the higher energy state in a VB move to the lowest energy states in the CB under the same point in the Brillouin zone after the electronic absorption process^52,53^. Therefore, by plotting and extrapolating a graph between [F(R) hυ]^n^ and hυ for n = 2 and 1/2, E_g_ values corresponding to the different concentrations of Ni-ion in BTFO samples are calculated by the linear portion of the curve (Fig. 7a and b). Figure 7c, the Eg values measured to be 1.021, 0.289, 0.289, and 0.289 eV for BTFO/xNiFO (x = 0.0 to 0.5) nanocomposites in the direct case, and 2.674, 1.482, 1.482, and 1.482 eV in the indirect case. This gap is due to the presence of intermediate energy levels for both the valence band (VB) and conduction band (CB). In the case of Ni-doped BTFO materials, the Eg values decrease with doping. The incorporation of Ni ion BTFO introduces ‘B-site’ vacancies, which contribute to structural deformation and distortion^49,53^. These vacancies of the ‘B site’ introduce shallow defects in the optical band gap and therefore the Eg value decreases. The B-site vacancies increase with the doping and resulting in the creation of the free carriers that have a many-body effect, which reduces electron energy as compared to a noninteracting carrier network. Such interaction can take the form of interaction between electron–electron, electron-donor, electron–hole, hole-hole, and hole-acceptor^49,53^.

The material refractive index is known to minimize with energy gap. Consequently, both these common quantities are thought to have a particular relationship. There were different efforts to locate an acceptable correlation (empirical and semiempirical) between the refractive index and the energy gap of semiconductors. Some of these attempts are the Moss relation and Vandamme relation^54,55^. The claimed relationships have already been presented with justifications for obtaining a satisfactory agreement to experimental results^54,55^ and so it will be used here to calculate the refractive index of the prepared samples from the obtained energy gaps.

**Figure 8:**
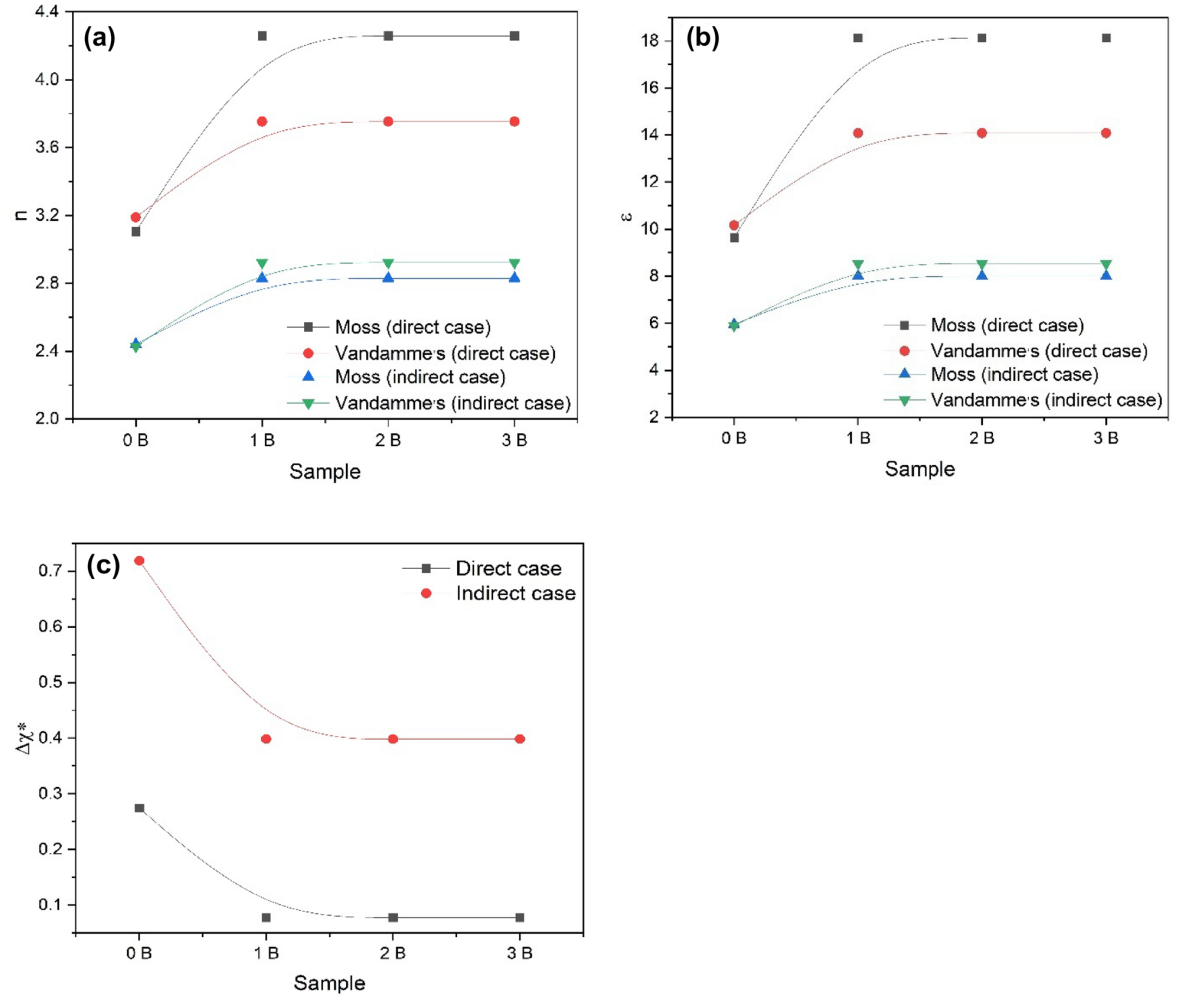
(**a**) Refractive index, (**b**) Optical dielectric constant, and (**c**) Electronegativity of BaTi_0.7_Fe_0.3_O_3_/(0, 1, 3, 5) NiFe_2_O_4_ nanocomposites in the wavelength range 200–2500 nm.

Figure 8a shows the change of the refractive index with Ni content calculated by different attempts described before for direct and indirect transition cases. It is obvious that there are small differences between the n values calculated by different methods and this may be due to the difference in the mathematical approximation methods used in every attempt. Also, the refractive index increases by NFO addition, and then the behavior is kept constant with NFO content increasing. This means that the prepared nanocomposites are desirable in applications that need a constant refractive index such as military applications, space, and optical devices.

The material dielectric constant is linked to the refractive index^54^ by ($\varepsilon_{\infty } = n^{2}$). The dielectric constants of the prepared samples are given in Fig. 8b for direct and indirect gap cases respectively. It was noted that the dielectric constant change behavior with Ni change is similar to the behavior of the refractive index.

Duffy^56^ has demonstrated the earlier view and presented it as optical electronegativity (Δχ*) and get the formula that links it by energy gap ($\Delta \chi^{*} = 0.2688 E_{g}$). The Duffy relation is used in calculating the optical electronegativity of the prepared samples for the direct and indirect transition gaps, and the resulting values were presented in Fig. 8c. The presented data shows that the BTFO/NiFO optical electronegativity lies between (0.7–0.07) for both direct and indirect cases, respectively. Following Pauling's^57^ indication, the current samples are covalent as predicted by XRD.

### Magnetic properties

**Figure 9:**
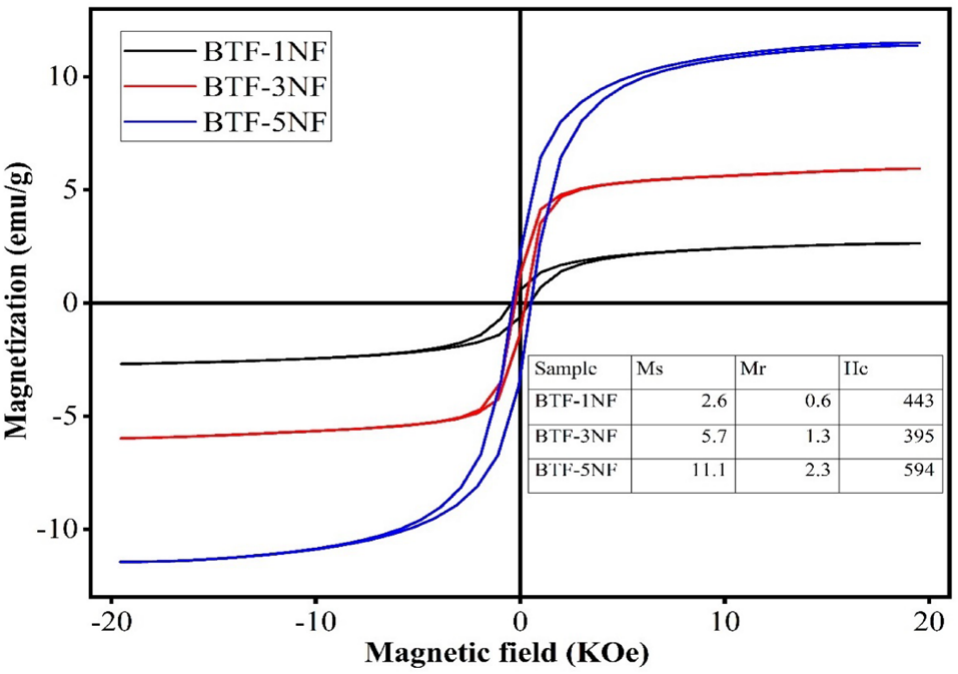
Magnetic hysteresis loops of iron barium titanate (BTF) shielded with nickel ferrite (BTF/xNFO).

Figure 9 shows the magnetic hysteresis loop of iron barium titanate shielded with nickel ferrite (nano-oval BaTi_0.7_Fe_0.3_O_3_ shielded (0.1, 0.2, 0.5) NiFe_2_O_4_) nanocomposites. The M–H curve of the composites exhibits a saturated magnetic hysteresis loop that the composites samples have a ferromagnetic nature. This hysteresis loop for all samples is small, which is a specific feature for a soft ferrite NiFe_2_O_4_.The composites gained their magnetic property from the presence of the ferromagnetic phase (NiFe_2_O_4_). The magnetic nature of NiFe_2_O_4_ nanopowder results from the antiparallel distribution of the magnetic centers (Ni^2+^ and Fe^3+^) between the octahedron and the tetrahedron sites. Therefore, increasing the concentration of the ferromagnetic phase (NF) in the composite increases the magnetic centers in the composite samples, and hence the total magnetic moment of the samples increases too. Therefore the saturation magnetization of the composites increases with increasing the NF concentration. The diamagnetic phase BTF decreases the interaction between the magnetic centers NF and hinders the rotation of the magnetic centers with the applied magnetic field, therefore the saturation magnetization decreases with increasing the diamagnetic phase concentration^58,59^. The magnetic parameters (saturation magnetization Ms, Remanent magnetization Mr, and coercive field Hc) of the composites are listed in the inset of Fig. 9 to highlight that increasing the ferrite content in the composite improves the magnetic properties of the composites. This can be explained on the basis of the presence of Ni^2+^ and Fe^3+^ ions present in the NiFe_2_O_4_ system as a coated layer. Since the increase of their contents may be causing the increase of their unit cell, thereby increasing the lattice parameter and exchange interactions within A and B sites. Also, the presence of Fe3 + in the based BaTi0.7Fe0.3O3 and in the NFO layer enhances the magnetization of the B-site.

### Dielectric (relative dielectric permittivity)

**Figure 10:**
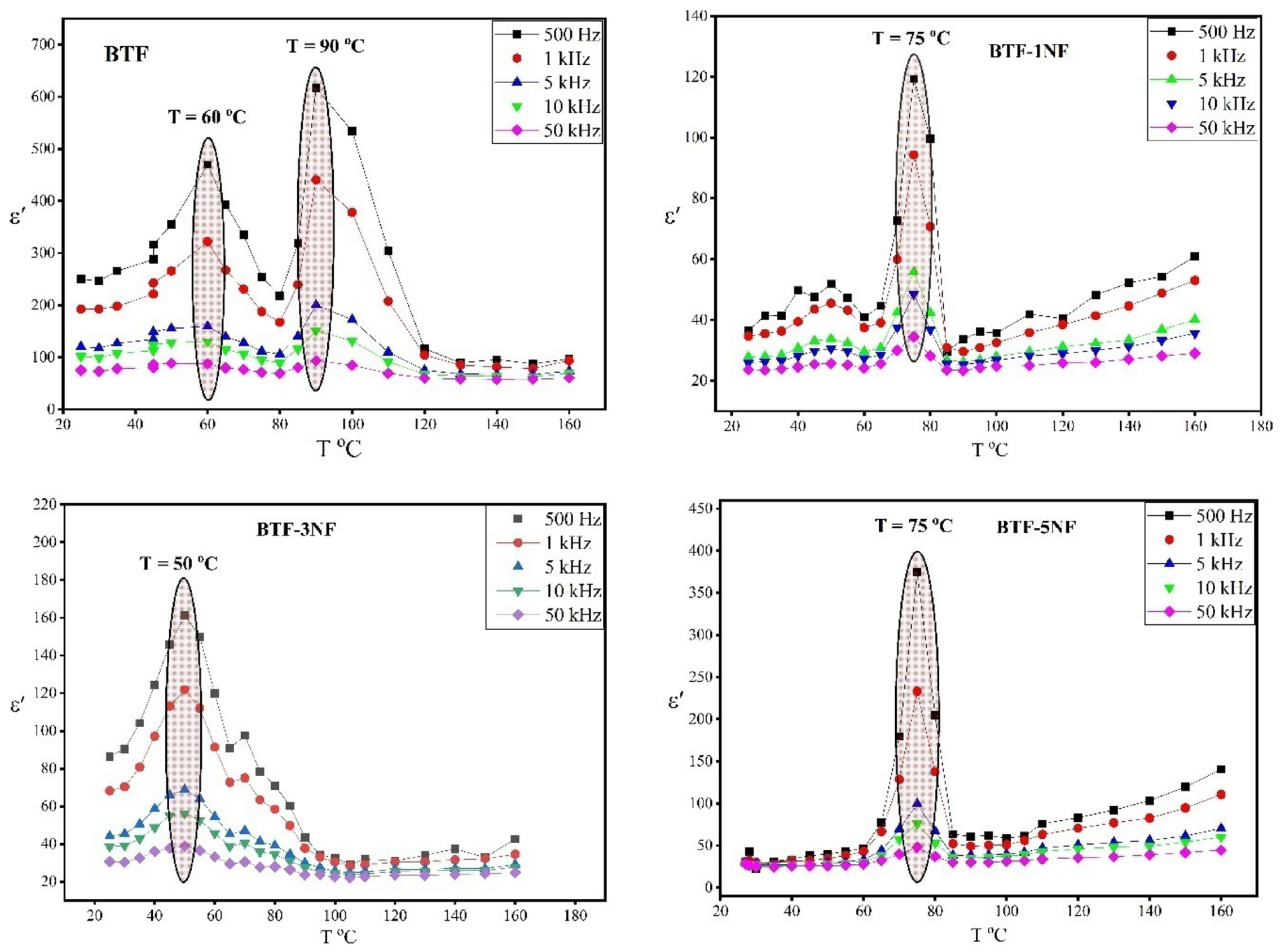
Relative dielectric permittivity (ε'(ν)) versus temperature at different frequency of iron barium titanate (BTF) and iron barium titanate shielded with nickel ferrite (BTF/xNFO).

The relative dielectric permittivity (ε'(ν)) of the composite samples (BTF/xNFO) against temperature at different frequencies is shown in Fig. 10. As a result, an increasing behavior in the dielectric permittivity with increasing temperature was obtained until a critical temperature. Then, a decrease in the dielectric permittivity was observed with higher temperatures. This critical temperature is the Curie temperature and represents the transition from the ferroelectric to the para-electric state. According to Fig. 10, the BTF sample has two transition temperatures; the first transition is assigned to the presence of an unstable monoclinic phase, while the second one represents the ferroelectric transition temperature.

### Electrochemical properties

**Figure 11:**
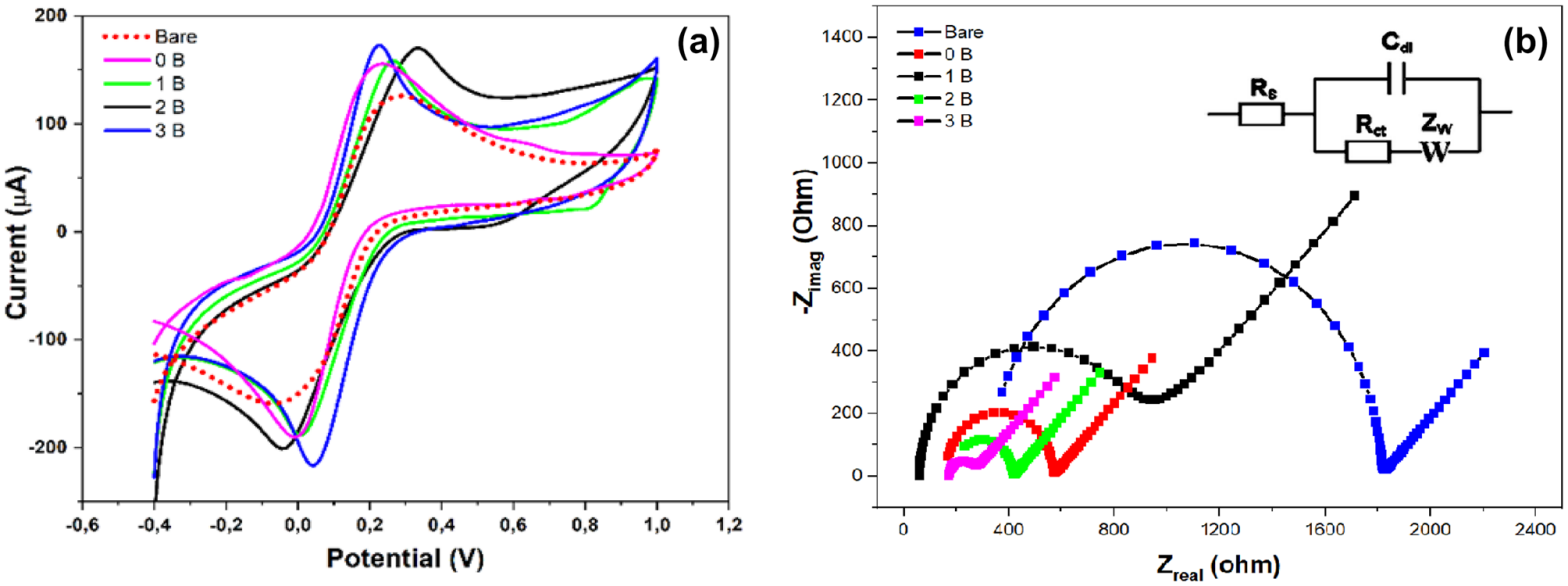
**(a)** CV measurements of (Bar, 0, 1,2 and 3 B) were conducted in a solution of standard redox probe of ferricyanide with the 5 mM in KCl as the supporting electrolyte. Scan rate of 50 mV s − ^1^ was applied for all experiments. (**b**) Show EIS Nyquist spectra characterization of modified electrodes with the nanomaterials. The inset represent the modeled Circuit thatis used for curve fitting.

Electrochemical behaviors of the prepared nanocomposite are identified, in a stranded redox mediator of ferro/ferricyanide, by cyclic voltammetry (CV) and electrochemical impedance spectroscopy (EIS). As a result, a fast oxidation–reduction and highest faradic current of the standard redox probe were obtained for all nanocomposite-based electrodes (0, 1, 2 and 3B), as presented in Fig. 11a and b. The ascending order in the voltammetric signals is as follows: 3B > 2B > 1B > 0B, as depicted in Fig. 11a and Table 2.Therefore, the highest conductivity alongside electrochemical activity is dedicated to the modification of electrode surface with the nanocomposite (3B).

**Table 2:** The electrochemical parameters (CV & EIS) obtained for the modified electrodes with the prepared nanomaterials.

Electrode type	*I*_*a*_ (µA)	*I*_*c*_ (µA)	E _*oxd*._ (V)	E_*red.*_ (V)	E_1/2_ (V)	R_s_ (Ω)	R_ct (1)_ (Ω)	C µF	W (Ω)
Bare	124	− 158	0.274	− 0.06	0.107	324	1400	0.136	0.0028
0 B	153	− 188	0.233	− 0.05	0.09	159	407	0.243	0.0023
1 B	159	− 189	0.268	0.01	0.139	184	231	0.432	0.02216
2 B	169	− 202	0.334	− 0.03	0.152	126	148	0.573	0.00227
3 B	171	− 216	0.227	0.045	0.136	173	88	1.231	0.0020

Those values are extracted from the above discussed voltammetric as well as the impedimetric experiments (Fig. 11).

In parallel to the voltammetric studies, EIS analysis was conducted for all modified electrodes, as displayed in Fig. 11b, and Table 2. Nyquist plots, at high frequency, showed a semicircle portion reflecting the changes in the electron transfer resistances, whereas the charge transfer resistance (R_ct_) at the electrode interface was presented by the diameter of the semicircle. Matching with the voltammetric results, the lowest resistances were obtained from 3B-based electrode (Rct = 88.4Ω) followed by 2B (R_ct_ = 148.7Ω), 1B (Rct = 231.2 Ω), and 0B (Rct = 407.8Ω). Worth mentioning here that the all composite-based electrodes provided lower charge transfer resistances than that obtained by the unmodified electrodes (Rct = 1400.8 Ω). The inset in Fig. 11b represents the equivalent circuit used for fitting the impedance Nyquist plots. From the CV and EIS results, we could conclude that the 3B composite has a promising property and can be exploited in electrochemical applications.

**Figure 12:**
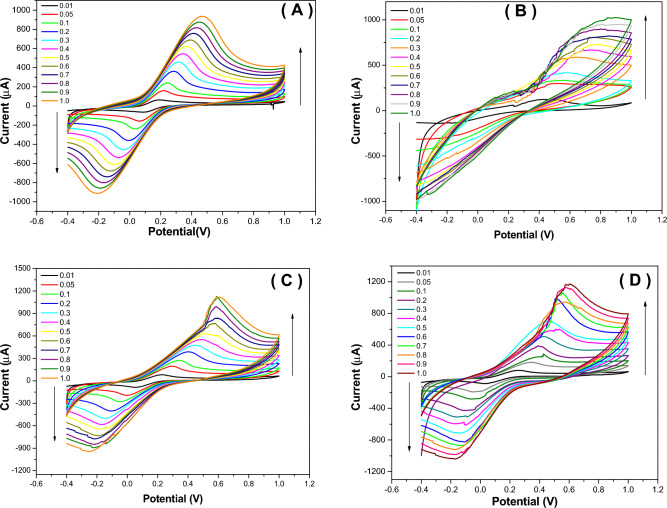
CV of prepared nanocomposites (**A**) 0B, (**B**)1B, (**C**)2B, and (**D**) 3B at different scan rates. Screen printed electrodes were modified with a thin film of the nanomaterials and voltammetric experiments were performed in the ferricyanide (5 mM).

**Figure 13:**
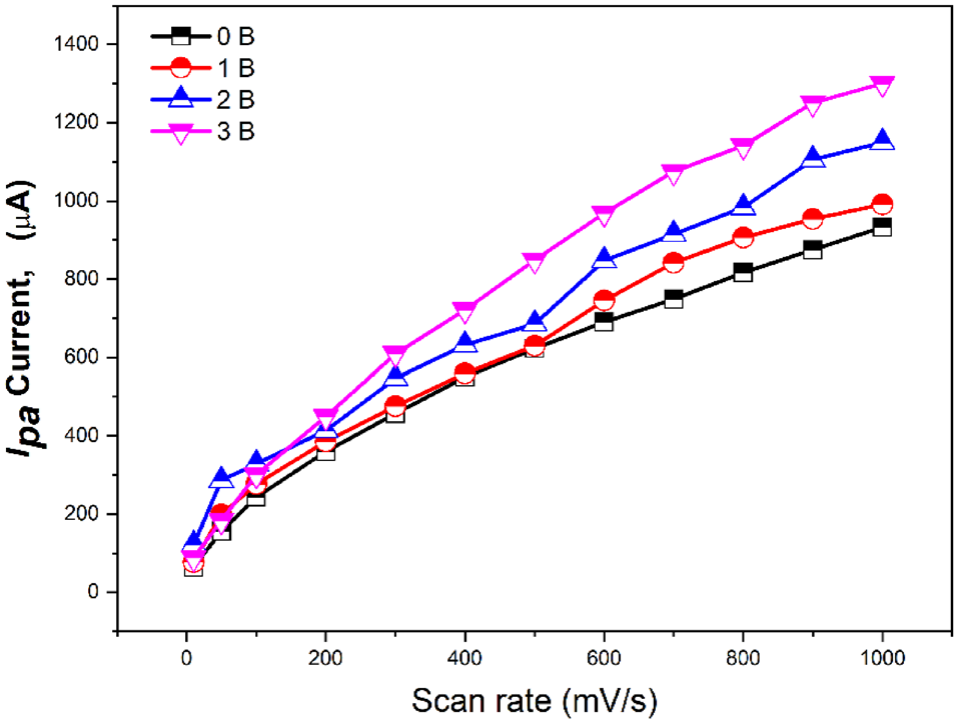
Influence of scan rate changes on the oxidation currents of the 0B, 1B, 2B and 3B nanostructure-based electrodes at different scan rate.

The redox peaks which appeared in the CV curves for prepared nanocomposites showed a pseudocapacitive electrode material significant feature. In the charge/discharge process, very fast reversible faradaic redox occurred along with the faradaic charge transfer and intercalation of protons at the surface of the electrodes. The effect of scan rate on the electrochemical behavior of each prepared nanomaterial (0, 1, 2, and 3B) was studied by the cyclic voltammetry method (Fig. 12A, B, C and D). In the scan rate range from 0.01 to 1.0 V/s, the redox peak currents (i_pa_ and i_pc_) increased as the scan rate increased for 0, 1, 2, or 3B modified electrodes. In Fig. 13, the lower electrical conductivity of 0B is due to lower peak current values. On the other hand, the values of peak current increased in 1B and 2B.The highest peak current values were observed in 3B, which refers to its capacitive properties. However, the cyclic voltammogram exhibited a well-known hysteresis-type loop, which is significant for a supercapacitor. Furthermore, all prepared nanomaterials were stable electrochemically over various applied scan rates, and no damage was produced in the electrode-modified composition.

### The use of 0B, 1B, 2B and 3B nanostructures for H_2_O_2_ detection

**Figure 14:**
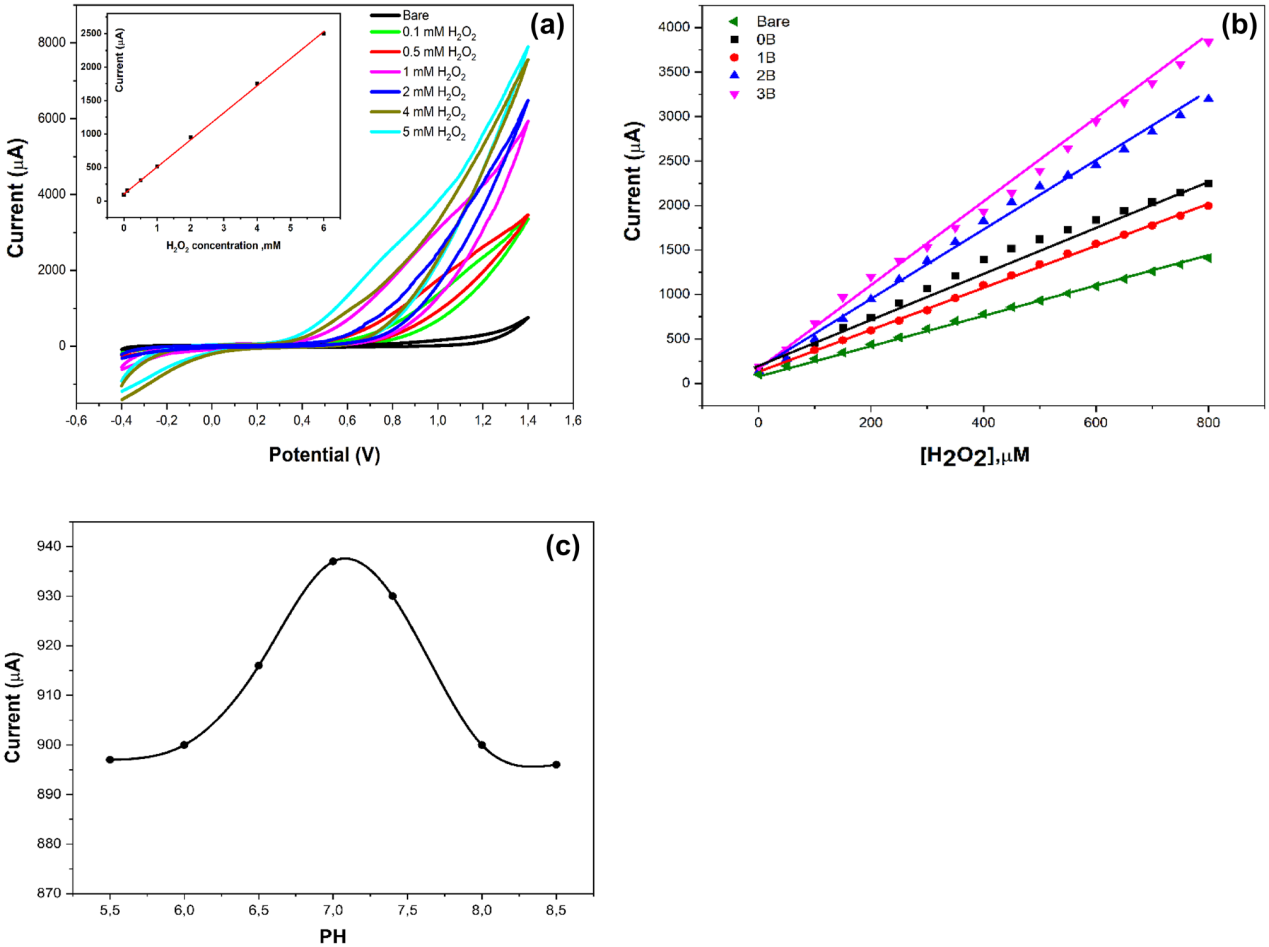
**(a)** Cyclic voltammety of 3B-based electrodes towards the direct voltammetric oxidations of different concentrations of H_2_O_2_ in (PBS pH = 7.0). The calibration curve is presented in the inset figure. (**b**) Chronoamperometric calibration curves of bare, 0B, 1B, 2B, and 3B toward different concentrations of H_2_O_2_ in (PBS pH = 7.0). (**c**) pH effect on the sensor performance towards the sensitivity of direct peroxides detection.

The electrochemical properties of nanocomposite materials modified electrode is one of the key benefits to supply high electrocatalytic activity which produces fast electron transfer and direct oxidation in the field of non-enzymatic biosensors. Therefore, the study of direct electron transfer produced from the oxidation of hydrogen peroxide evaluated overall prepared nanomaterials. The direct oxidation was measured by CV and chronoamperometric methods by adding different peroxides concentrations into the electrochemical cell. The cyclic voltammetry graph and a calibration curve are represented in Fig. 14a and chronoamperometric calibration curves of bare, 0B, 1B, 2B, and 3B modified electrodes are shown in Fig. 14b. The highest signals were obtained by the 3B modified electrode. Therefore, the high electrocatalytic activity of 3B allowed the direct electrochemical oxidation of peroxide. In addition, as the concentration of H_2_O_2_ increased, an increase in oxidation peak currents were produced which revealed the high sensitivity and reliability of the 3B modified SPE.

Consequently, the amperometric signal of peroxide oxidation at different pHs was studied. As represented in Fig. 14c), the amperometric response of the 3B-based electrodes towards the peroxide’s oxidation was measured at different pHs, and the electrochemical signal increased as the pH increased from 4 up to 7 then decreased above pH = 7.0. So that, PBS buffer with pH 7.0 was selected for all subsequent experiments.

### Amperometric detection of peroxide

**Figure 15:**
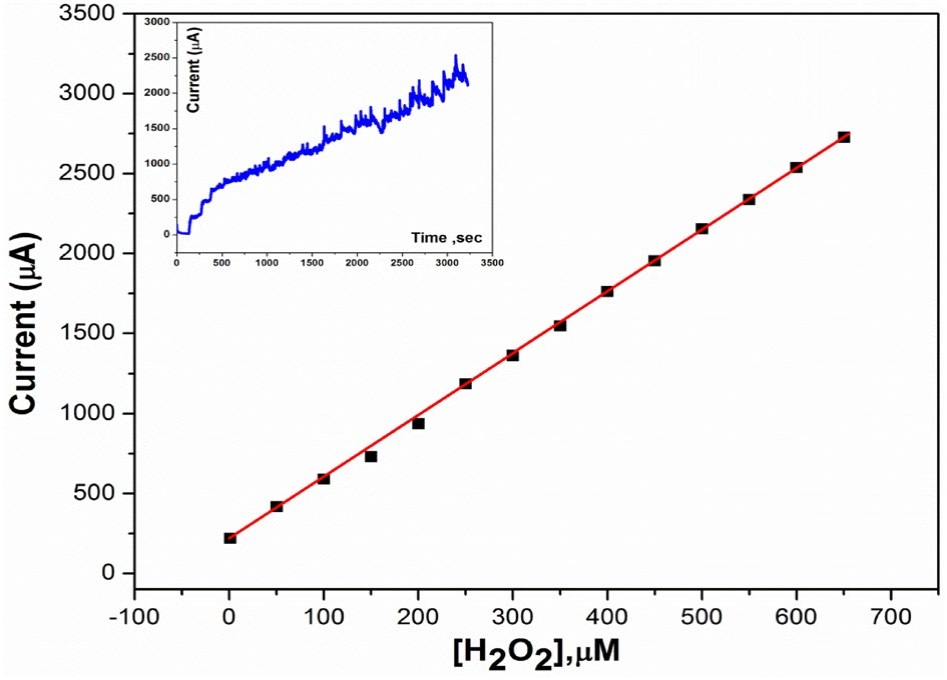
The corresponding calibration curve toward H_2_O_2_ response.

From cyclic voltammetry (CV) detection, the peroxide oxidation peak was produced at 0.7 V. Therefore, the amperometric study was evaluated at 0.7 V by adding a standard concentration of H_2_O_2_ at a fixed time (100 s). Figure15 showed the relation between the different concentrations of peroxides and current response with a fast and high response, which approved the fast electron transfer due to the electro-catalytic behavior of the 3B-based electrodes. The calibration curve (as it is clear in Fig. 15) showed a linear range from 0.1 up to 650 µM with a detection limit of 0.01 µM, which approved the high sensitivity of the proposed electrode to be effectively applied in the non-enzymatic-based biosensors field. Additionally, a comparison of electrochemical response between electrochemical performance of the newly developed electrode for peroxide determination and the other reported materials is tabulated in Table 3.

**Table 3:** Comparison of the analytical performance of various Hydrogen Peroxide Sensors (with various literatures).

Electrode	Linear range (µM)	Detection limit (µM)	Applied potential (V)	Ref
Poly(azureA)PtNPs/SPCE	0–300	0.052	0.1	^60^
Co_3_O_4_-rGO	15–675	2.4	− 0.19	^61^
rGO-Pt	0.5–3475	0.2	− 0.08	^62^
Pt/rGO-CNT paper electrode	0.1–25	0.01	− 0.25	^63^
Co_3_O_4_/SPCE	0.1–50	0.145	1.0	^64^
MnCo_2_O_3_/CNTs/SPE	0.1–180	0.1	0.7	^24^
BaTi_0.7_Fe_0.3_O_3_@NiFe_2_O_4_	0.1–650	0.01	0.70	This work

*Screen printed carbon electrode *SPCE*, Screen-printed electrode *SPE*, *NPs* Nano plates.

## Conclusion

From this work, activated NiFe_2_O_4_ nano-oval was successfully shielded with BaTi_0.7_Fe_0.3_O_3_ nanoperovskite synthesized via the sol–gel chemical manner and calcined at 600 °C. The XRD powder asserted the formation of bi-phases for pero-NiFe_2_O_4_ nanocomposites. The measured band gap energy was 1.021, 0.289, 0.289, and 0.289 eV in the direct case, and 2.674, 1.482, 1.482, and 1.482 eV in the indirect case, respectively for BTFO/xNiFO (x = 0 to 5) nanocomposites. The incorporation of Ni ion into BTFO introduces ‘B-site’ vacancies, which contribute to structural deformation and distortion. . With the addition of Ni ion, the electronegativity exhibits a behavior similar to that of the band gap, but the refractive index and optical dielectric constant exhibit opposite behavior. The addition of NiFe_2_O_4_ plays an appreciable role in enhancing temperature stability and increasing the permittivity of the pero-magnetic BaTi_0.7_Fe_0.3_O_3_@ NiFe_2_O_4_ nanocomposites. The pero-magnetic BaTi_0.7_Fe_0.3_O_3_@NiFe_2_O_4_ nanocomposite electrode has been used as a sensing material for H_2_O_2_ detection. The results of electrochemical data approved that the new BaTi_0.7_Fe_0.3_O_3_@NiFe_2_O_4_ nanocomposites can be used effectively to direct H_2_O_2_-detection in biological analysis such as enzymatic-based sensors. Eventually, the obtained results are of excessive value to progress the structural, morphological, thermal, electrical, and electrochemical properties of pero-nanomagnetic composites at different calcination and ferrites contents, which enable be projected to apply in various applications as catalytic, biosensors, electromagnetic interference shielding systems, …. etc.

## Supplementary Information

Supplementary Information 1.

Supplementary Information 2.

Supplementary Information 3.

Supplementary Information 4.

Supplementary Information 5.

Supplementary Information 6.

Supplementary Information 7.

Supplementary Information 8.

## Data Availability

Additional information Correspondence and material requests should be addressed to A.M. El Nahrawy (amany_physics_1980@yahoo.com. am.elnahrawy@nrc.sci.eg).
